# Supplementary material for: A Recombinant Protein XBB.1.5 RBD/Alum/CpG Vaccine Elicits High Neutralizing Antibody Titers against Omicron Subvariants of SARS-CoV-2
Source: Vaccines (Basel). 2023 Oct 1;11(10):1557. doi: 10.3390/vaccines11101557 (PMC10610638; doi:10.3390/vaccines11101557)
Supplement: Supplementary file 1 [file vaccines-11-01557-s001.zip › vaccines-2617021-supplementary.pdf]

Supplementary Materials:

Table S1: Recombinant RBD proteins evaluated in this project.

RBD lot# used for vaccination	Purification ID	Fermentation ID	RBD classification
S2RBD-150121JXL-1	PDD011221	PDF110220C	WT
S2RBD-140421JXL-1	PDD041221	PDF032221B	Beta
S2RBD-300721JXL-1	PDD072721A	PDF071221A	Delta
S2RBD-141022JXL-1	PDD101022	PDF082922B	BA.4/5
Sc2RBD-140423JXL-1	PDD041123	PDF032023B1	XBB.1.5

List of mutations included in the SARS-COV-2 spikeprotein of the lentiviral-based pseudoviruses

BA.2.75.2: T19I, del24-26, A27S, G142D, K147E, W152R, F157L, I210V, V213G, G257S, G339H, R346T, S371F, S373P, S375F, T376A, D405N, R408S, K417N, N440K, G446S, N460K, S477N, T478K, E484A, F486S, Q498R, N501Y, Y505H, D614G, H655Y, N679K, P681H, N764K, D796Y, Q954H, N969K, D1199N

BA.4: T19I, del24-26, A27S, del69-70, G142D, V213G, G339D, S371F, T376A, D405N, R408S, K417N, N440K, L452R, S477N, T478K, E484A, F486V, Q498R, N501Y, Y505H, D614G, H655Y, N679K, P681H, N764K, D796Y, Q954H, N969K

BQ.1.1: T19I, del24-26, A27S, del69-70, G142D, V213G, G339D, R346T, S371F, T376A, D405N, R408S, K417N, N440K, K444T, L452R, N460K, S477N, T478K, E484A, F486V, Q498R, N501Y, Y505H, D614G, H655Y, N679K, P681H, N764K, D796Y, Q954H, N969K

XBB.1.5: T19I, L24S, del25-27, V83A, G142D, del144, H146Q, Q183E, V213E, G252V, G339H, R346T, L368I, S371F, S373P, S375F, T376A, D405N, R408S, K417N, N440K, V445P, G446S, N460K, S477N, T478K, E484A, F486P, F490S, Q498R, N501Y, Y505H, D614G, H655Y, N679K, P681H, N764K, D796Y, Q954H, N969K

XBB.1.16: T19I, L24S, del25-27, V83A, G142D, del144, H146Q, E180V, Q183E, V213E, G252V, G339H, R346T, L368I, S371F, S373P, S375F, T376A, D405N, R408S, K417N, N440K, V445P, G446S, N460K, S477N, T478R, E484A, F486P, F490S, Q498R, N501Y, Y505H, D614G, H655Y, N679K, P681H, N764K, D796Y, Q954H, N969K

EG.5.1: T19I, L24S, del25-27, Q52H, V83A, G142D, del144, H146Q, Q183E, V213E, G252V, G339H, R346T, L368I, S371F, S373P, S375F, T376A, D405N, R408S, K417N, N440K, V445P, G446S, F456L, N460K, S477N, T478K, E484A, F486P, F490S, Q498R, N501Y, Y505H, D614G, H655Y, N679K, P681H, N764K, D796Y, Q954H, N969K

Fitted data plots of pseudovirus neutralization experiments

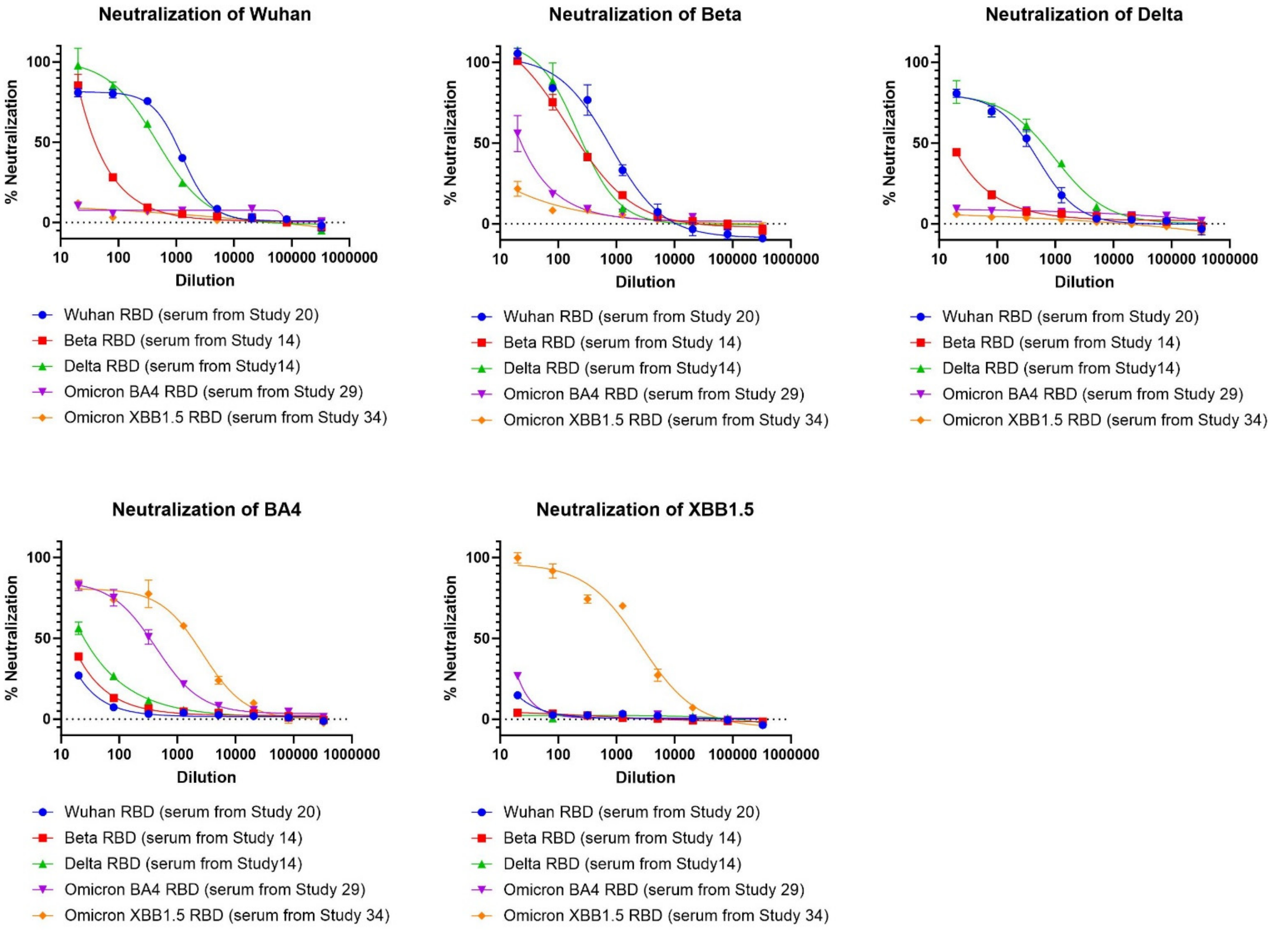

Figure S1. Neutralizing antibody titers of sera from mice immunized with five variant RBDs (WT, Beta, Delta, BA.4/5 and XBB.1.5) adjuvanted with Alum+CpG against a panel of five pseudovirus variants (WT, Beta, Delta, BA.4/5 and XBB.1.5). Cross-protection against pseudoviruses decreases progressively from WT RBD to XBB 1.5. (Varying levels of cross-protection among WT, Beta, and Delta, but almost none against Omicron BA 4/5 and XBB 1.5). On the other hand, XBB 1.5 antigen showed cross-protection against Omicron BA 4/5, while BA 4/5 RBD showed very low-level neutralization titers against XBB 1.5 pseudovirus.
